# Accidental Toothbrush Ingestion

**DOI:** 10.7759/cureus.62955

**Published:** 2024-06-23

**Authors:** Abhijeet Karad, Kranthi Dandi, Debabrata Banerjee, Amol S Dahale, Yogesh Bade

**Affiliations:** 1 Department of Gastroenterology and Hepatology, Dr. D.Y. Patil Vidyapeeth, Pune, IND

**Keywords:** accidental ingestion, gastroenterology, gastrointestinal foreign body, endoscopy, toothbrush

## Abstract

The accidental ingestion of a toothbrush is an extremely rare occurrence, typically involving young women with psychiatric disorders such as schizophrenia, bulimia, or anorexia nervosa. There are no known cases of a swallowed toothbrush being expelled naturally through the rectum. Therefore, prompt extraction of an ingested toothbrush from the gastrointestinal tract using a surgical or endoscopic method is a necessity. Here, we report a case of a psychologically healthy woman ingesting a toothbrush accidentally while cleaning her tongue with the back of the toothbrush. In our report, we document the successful extraction of a toothbrush from the esophagus endoscopically without any complications.

## Introduction

Ingestion of foreign objects in the gastrointestinal tract occurs most frequently in children between six months and three years of age, accounting for 80% of such incidents [1]. Children's innate curiosity often leads them to explore objects by mouth, which can result in swallowing them. Coins are the most commonly swallowed foreign objects followed by marbles, safety pins, bottle tops, and button batteries [2,3].

In adults, foreign body ingestion may occur accidentally or intentionally, especially in individuals with psychiatric conditions such as bulimia nervosa, schizophrenia, or suicidal tendencies. The management of ingested foreign bodies is highly individualized, taking into account various factors such as the patient's age, symptoms, and overall health as well as the type, location, and orientation of the object within the gastrointestinal tract. Toothbrush ingestion, though rare, necessitates immediate medical intervention as it is improbable for the toothbrush to pass through the gastrointestinal tract on its own, particularly the "C" loop of the duodenum, due to its shape and length [4].

## Case presentation

**Figure 1:**
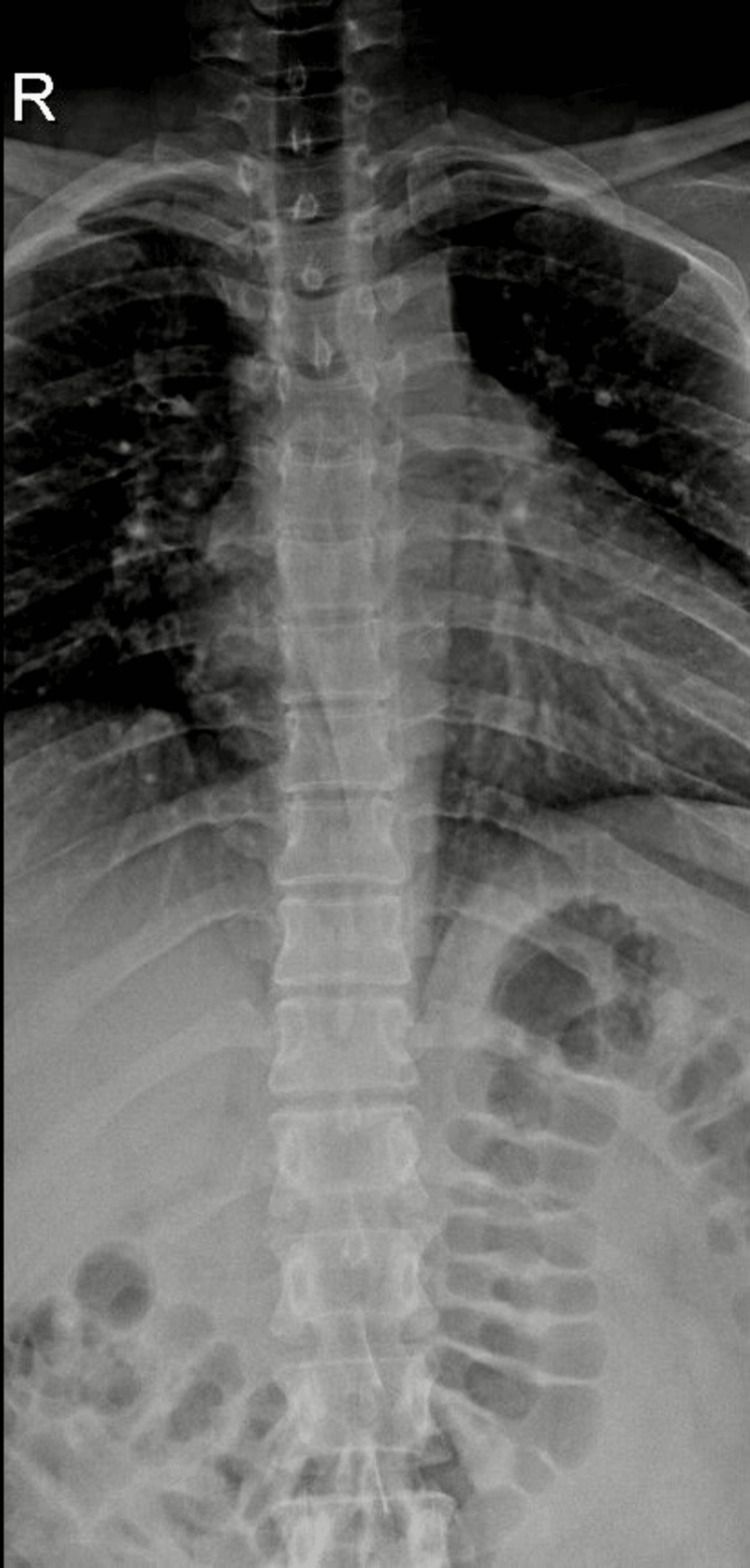
X-ray of the chest and abdomen

A 42-year-old woman with no previous comorbidities self-reported the accidental swallowing of a toothbrush. She narrated that the incident took place while she was utilizing the reverse side of the brush head to clean the back portion of her tongue. She didn't experience any chest pain, abdominal pain, or breathlessness following the ingestion of the toothbrush. However, out of anxiety, she self-reported this incident to healthcare providers, and within an hour of this incident, she reported to the Medical Gastroenterology Department of our hospital. On examination, she had stable vital parameters with a pulse rate of 87 beats per minute, blood pressure of 110/70 mm Hg, and an oxygen saturation of 98% in room air. The systemic examination yielded no significant findings. A roentgenogram of the chest and abdomen revealed no radiopaque object in the gastrointestinal tract (Figure 1). The patient's laboratory tests did not show any significant abnormalities (Table 1).

**Table 1:** Laboratory investigations AST, aspartate transaminase; ALT, alanine transaminase; ALP, alkaline phosphatase; TIBC, total iron binding capacity; total T3, total triiodothyronine; total T4, total tetraiodothyronine; TSH, thyroid stimulating hormone; g/dl, grams/deciliter; /μL, /microliter; mmol/L, milli moles/liter; mg/dl, milligrams/deciliter; U/L, units/liter; μg/dl, micrograms/deciliter; μIU/ml, micro international units/milliliter.

Laboratory investigations	Patient's report	Reference ranges
Hemoglobin	12.4 g/dl	11.6-15 g/dl
Total leukocyte count	9,200 /μL	4,000–10,000 /μL
Platelet count	3,17,000 /μL	1,50,000–4,10,000 /μL
Sodium	139 mmol/L	136–145 mmol/L
Potassium	3.59 mmol/L	3.50–5.10 mmol/L
Chloride	99 mmol/L	98–107 mmol/L
Urea	18 mg/dl	17-49 mg/dl
Creatinine	0.70 mg/dl	0.6-1.2 mg/dl
Total bilirubin	0.31 mg/dl	0.22-1.20 mg/dl
Direct bilirubin	0.20 mg/dl	Upto 0.50 mg/dl
AST	24 U/L	8-43 U/L
ALT	20 U/L	7-45 U/L
ALP	64 U/L	35-104 U/L
Total proteins	7.30 g/dl	6.4-8.3 g/dl
Albumin	4.50 g/dl	3.50-5.20 g/dl
Iron	65 mg/dl	50-150 mg/dl
TIBC	373 μg/dl	250-450 μg/dl
Transferrin saturation	24%	20-50%
Ferritin	198 ng/ml	4.63-204 ng/ml
Total T3	0.72 ng/ml	0.64-1.52 ng/ml
Total T4	4.94 μg/dl	4.87-11.72 μg/dl
TSH	1.16 μIU/ml	0.35-4.94 μIU/ml

The patient was counseled about the procedure of endoscopy extraction of an ingested toothbrush and the potential complications of the procedure.

**Figure 2:**
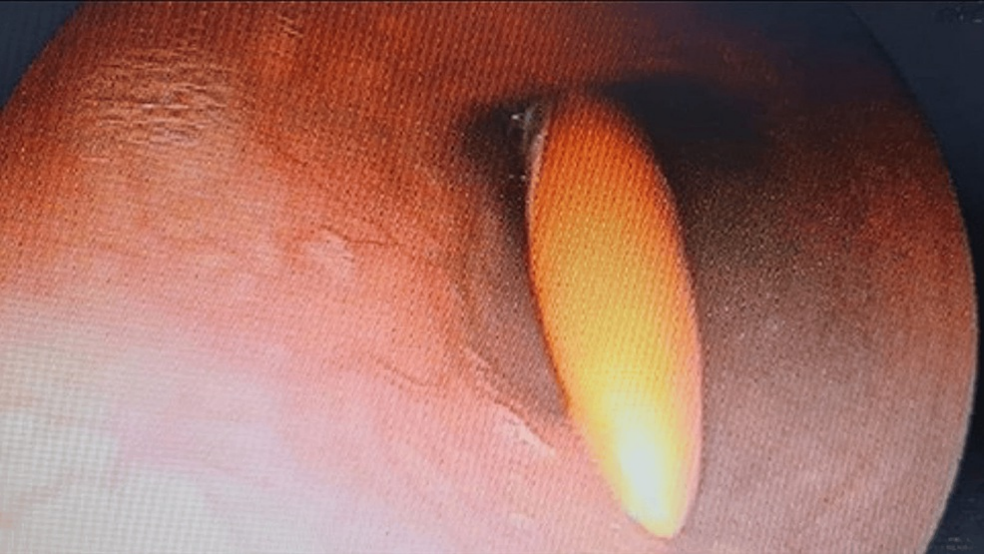
Tail end of toothbrush in esophagus.

**Figure 3:**
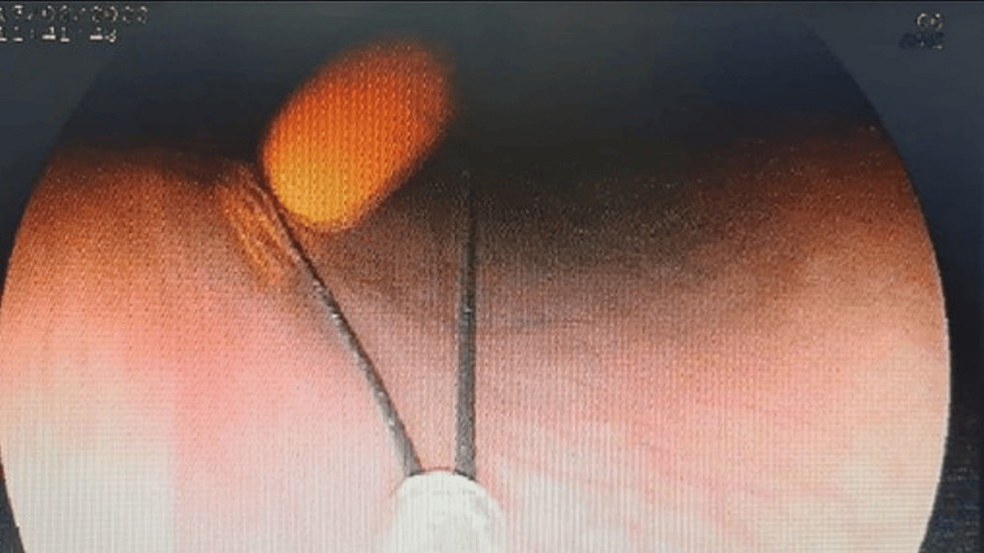
Toothbrush being extracted using a snare.

**Figure 4:**
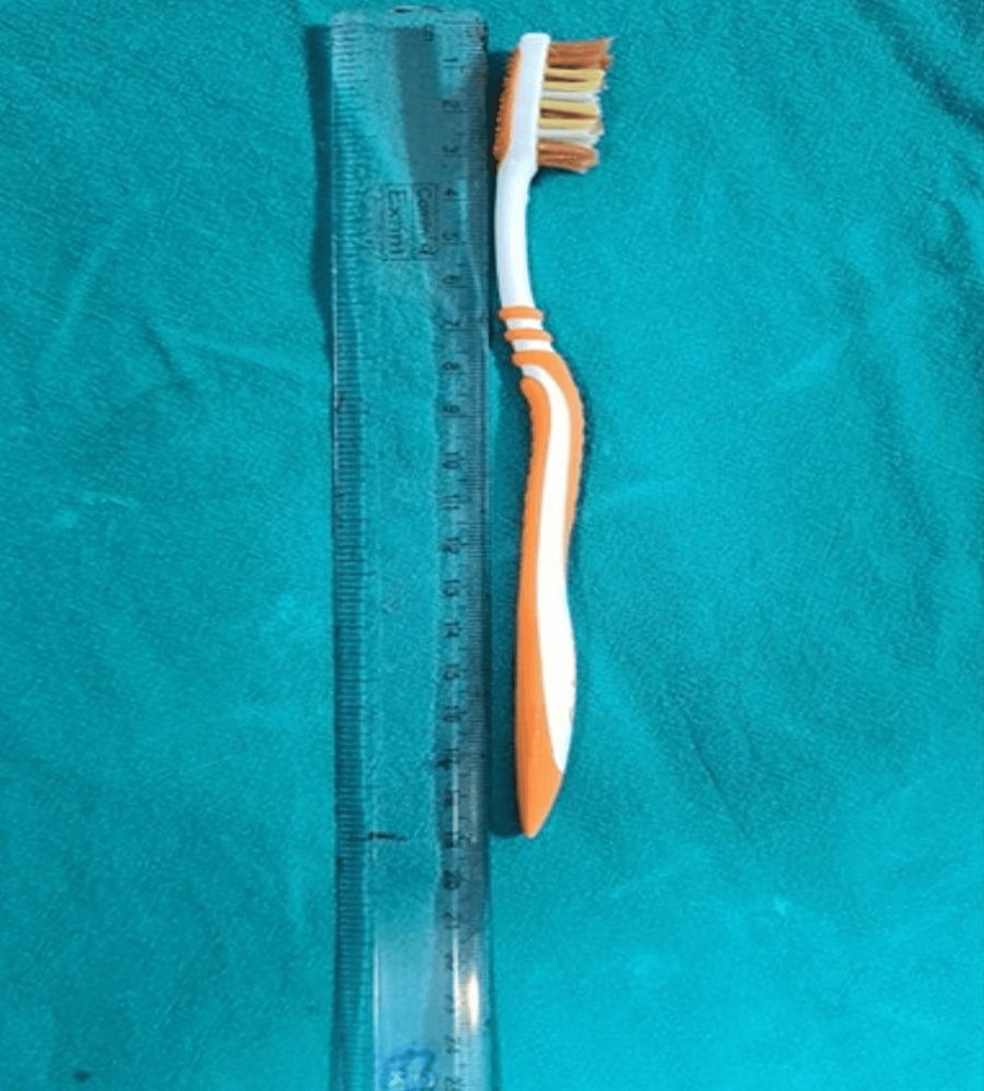
Length of toothbrush measured to be 19 cm.

Following informed consent, an upper gastrointestinal endoscopy was carried out after applying two puffs of 10% lignocaine spray per orally to the oropharynx. The toothbrush's tail was found approximately 5 cm distal to the upper esophageal sphincter (Figure 2). The toothbrush was aligned with the esophagus's long axis, within its lumen. Further passage of the scope could have pushed the toothbrush into the stomach, thereby complicating its extraction. Therefore, we decided to extract the toothbrush endoscopically, even before visualizing the entire toothbrush. Under endoscopic visualization, an endoscopic snare (35 mm; Medorah Meditek, Gurugram, India) was passed through the scope's working channel up to the waist of the toothbrush, and there it was held (Figure 3). Slowly, the toothbrush was pulled into the oropharynx. Once the tail of the toothbrush was visualized in the oral cavity of the patient, it was held by the endoscopist's fingers, and the patient's neck was hyperextended, allowing the removal of the toothbrush without any injury to the oropharynx. A relook endoscopy revealed no signs of esophageal laceration, perforation, or significant mucosal damage. The patient was monitored for 48 hours after the extraction. The length of the extracted toothbrush was 19 cm (Figure 4).

## Discussion

Foreign body ingestion is common among children, individuals with intellectual disabilities, psychiatric conditions, alcohol dependency, and elderly people who use dental prosthetics [4-6]. Usually, 80-90% of swallowed foreign bodies successfully pass through the gastrointestinal system without any intervention [7]. Endoscopic retrieval is necessary in 10-20% of the cases, while surgery is necessary to remove foreign bodies or address any problems in less than 1% of cases [8,9]. The impacted foreign body can lead to pressure necrosis, gastritis, mucosal tears, perforation, subcutaneous emphysema, and bleeding.

Toothbrush swallowing is rare. The ingestion of linear foreign objects exceeding 6-10 cm in length, like toothbrushes, presents a distinct challenge as their length prevents them from navigating the curvature of the duodenum, which is anchored by its fixed retroperitoneal attachment. No cases of spontaneous passage of the swallowed toothbrush were reported. The first reported death from a toothbrush occurred in 1889 as a result of gastric perforation three days after ingestion [10]. Kirk et al. reported 31 cases of toothbrush ingestion; none of them had spontaneous passage through the rectum [4].

Lee et al. reported a case of a 20-cm-long ingested toothbrush, traversing the pylorus, duodenal loop, and ileocecal valve, subsequently perforating the proximal transverse colon and penetrating the liver [11]. A laparotomy was performed, and the perforated organs were repaired.

Treatment methods for ingested foreign objects are constantly advancing. Previously, an emergency laparotomy was performed to retrieve the items and avert perforation. Nowadays, an initial approach often begins with endoscopy. Ertan et al. made the first successful attempt to retrieve a toothbrush using the endoscopy technique in 1983 [12].

Laparoscopic intervention is an option when endoscopic removal fails. Indeed, the laparoscopic method can serve as an alternative to laparotomy. Wishner and Rogers documented the inaugural successful laparoscopic extraction of an ingested toothbrush in 1997 [13]. Therefore, we recommend endoscopic removal of ingested toothbrushes at the earliest possible juncture and performing a laparoscopic gastrostomy for failed endoscopic retrieval.

While extracting a toothbrush, a special maneuver is necessary when it reaches the upper esophageal sphincter. The patient should be instructed to perform continuous swallowing movements to help relax the sphincter, allowing for easier removal. This is feasible only if the patient is under conscious sedation. It's crucial to maintain the toothbrush's long axis parallel to the esophagus during extraction. Additionally, when the toothbrush reaches the oropharynx, positioning the patient's head in extension before extracting the toothbrush can prevent injury to the oropharynx.

In our case, the patient self-reported the incident and sought medical consultation immediately, resulting in endoscopic intervention within four hours, which aided in a successful intervention without major complications. To prevent complications during the procedure, it is imperative to have a proficient team specializing in endoscopic interventions, a readily available endotracheal intubation tray, an anesthesiologist, and surgeons prepared to assist if needed. Surgical facilities equipped for laparotomy or laparoscopic intervention should ideally conduct the removal if endoscopy fails.

## Conclusions

The inadvertent ingestion of an entire toothbrush by an individual without an underlying mental health condition is extremely rare. However, such an event may happen unintentionally while attempting to clean the tongue with the head of the toothbrush. This report outlines the swift identification and decisive intervention by our skilled gastrointestinal endoscopy team, which resulted in the successful extraction of the toothbrush without any adverse outcomes.
